# How does ego depletion reduce sports performance in athletes? A systematic meta-analysis

**DOI:** 10.3389/fpsyg.2025.1528263

**Published:** 2025-06-16

**Authors:** Xu Yan, Jingyi Zhong, Yezheng Wang, Ying Tian, Lijuan Wang, Congcong Li, Kaihang Liu, Wei Zhao, Liyan Wang, Hongbiao Wang

**Affiliations:** ^1^Department of Physical Education, Shanghai University of Medicine and Health Sciences, Shanghai, China; ^2^School of Physical Education Science, Shenyang Normal University, Shenyang, China; ^3^College of Rehabilitation Sciences, Shanghai University of Medicine and Health Sciences, Shanghai, China

**Keywords:** ego depletion, sports performance, self-control, self-control force model, initiation task

## Abstract

**Purpose:**

This meta-analysis focused on exploring whether ego depletion affects sports performance. Subgroup analyses were conducted to compare the magnitude of effect sizes between different ego depletion initiation tasks and which type of sports performance is more susceptible to ego depletion as a moderator variable.

**Methods:**

This article was based on Pubmed, Web of Science, Scopus, EBSCO, Embase, and Cochrane databases for the included articles, and meta-analysis of the included articles was performed using RevMan 5.4 software to evaluate the effect of ego depletion on athletes’ sports performance through standardized mean difference.

**Results:**

Eleven articles and 12 studies were finally included. After sensitivity analyses using the Leave-One-Out method, two articles and one experiment were excluded with significant effect sizes. The final total effect size of ego depletion on athletes’ sports performance SMD = −0.38 [95% CI: −0.56 to −0.21], *P* = 0.001, demonstrating that ego depletion can produce a decrease in athletes’ sports performance. Subgroup meta-analysis showed that the Stroop task SMD = 0.63 [95% CI: −0.96 to −0.26] produced larger effect sizes than the transcription task SMD = 0.39 [95% CI: −0.64 to −0.13], i.e., the Stroop task was more likely to produce ego depletion in athletes. Targeting sports performance SMD = 0.49 [95% CI: −0.74 to −0.23] produced larger effect sizes than endurance-based sports performance SMD = 0.42 [95% CI: −0.68 to −0.16], i.e., aiming-based sports performance was more affected by ego depletion.

**Conclusion:**

The total effect size produced by ego depletion on athletes’ sports performance was decreasing, a moderate effect size, and there may be publication bias. The subgroup analyses showed that the amount of effect produced by different ego depletion initiation tasks was different and the Stroop task was more likely to be produced. Also, the effect sizes affected by ego depletion were different for various types of sports performance, with more pronounced for aiming movements.

**Systematic review registration:**

https://www.crd.york.ac.uk/PROSPERO/, identifier CRD42024561990.

## 1 Introduction

Athletes often face multiple pressures during the competition (spectator expectations, outcome uncertainty, etc.), which can easily trigger anxiety and thus affect athletic performance (Englert and Bertrams, 2015). Self-control, as a core competency, can help athletes manage emotions, maintain focus, and sustain perseverance, especially in adversity (Stocker et al., 2018). Daily high-intensity training is a process of self-control, and physical fatigue induces the urge to give up when stronger self-control is needed to enhance performance (Dorris et al., 2012). If long-term self-control fails, it will lead to ego depletion. In previous studies, researchers generally agree that the self-control strength model is one of the best theoretical models to explain the mechanism of action of ego depletion (Baumeister et al., 1998). Ego depletion is a concept in the field of psychology, which means that A state of ego depletion of psychological resources occurs when an individual experiences a decline in the psychological resources used after completing a self-control task (Baumeister et al., 1998).

### 1.1 Self-control power model and ego depletion

The power model of self-control posits that self-control is a limited psychological resource (Baumeister et al., 1998) whose ego depletion leads to decreased performance on subsequent tasks. This process is influenced by the dynamic interaction of intrinsic (cognitive, motivational) and extrinsic (environmental, social) factors. Researchers often test the theory in a dual-task paradigm: the experimental group first completes a self-control task (e.g., emotional suppression), the control group performs a neutral task, and the two groups are subsequently compared by irrelevant tests (e.g., physical fitness tests) (e.g., Stocker et al., 2018, found that the experimental group’s plank support time was shorter). Although Meta-analyses have been conducted to support the model (Giboin and Wolff, 2019; Hagger et al., 2010), there is still a gap in research on how the athlete population is affected by ego depletion, and the specific associations between self-control resource depletion and athletic performance in this population need to be further explored.

### 1.2 Ego depletion in athletes in the sports field

However, ego control theory models this conclusion and faces more complex challenges in competitive sports scenarios. Existing research suggests that ego depletion may trigger a decline in athletic performance (Dorris et al., 2012). This state has been shown to impair subsequent task performance across the domains of athletic competition (Fischer et al., 2012), risky decision-making, occupational performance (O’Brien et al., 2021), and withdrawal behaviour (Shmueli and Prochaska, 2012). For the athlete population, however, there is a lack of Meta-analytic evidence with clear effect sizes. This is because, in competitive play, athletes who develop ego depletion before a game can have serious consequences for subsequent play: sprinters can be disqualified for a rush violation (Englert and Bertrams, 2014; Englert and Bertrams, 2015), and basketball players can affect the game-winner when making critical free throws (Englert and Bertrams, 2012; Wagner and Wieczorek, 2024). Therefore, the amount of effect that ego depletion brings to athletes’ motor skills in the field of sports urgently needs to be calculated by meta-analysis methods, so that athletes and coaches will pay attention to the serious consequences of ego depletion and look for solutions.

### 1.3 Moderating variables of ego depletion in athletes in the field of sports

In previous ego depletion studies, researchers have preferred to study changes regarding moderating variables rather than overall effects (Hagger et al., 2010). The main moderating variables of ego depletion that researchers are interested in are the two moderating variables of different ego depletion initiation tasks and different types of sports performance (Giboin and Wolff, 2019).

### 1.4 Different ego depletion startup tasks

The types of initiation tasks in which researchers have used ego depletion include (1) Stroop tasks: subjects are asked to read only the colors written in the color words, ignoring the meaning of the color words themselves (Wagner and Wieczorek, 2024; Mangin et al., 2021; Dallaway et al., 2023); (2) transcription task: performing a task in which subjects are made to need to overcome their previous writing habits and achieve ego depletion by reducing the number of strokes in the writing process (Englert and Bertrams, 2012; Furley et al., 2013; Englert and Bertrams, 2016; Gregersen et al., 2017; Stocker et al., 2018). (3) Counting down numbers and keeping them a balanced task: counting down from 1000 in units of 7 or 5 and keeping the bubble level balanced in the same way (Dorris et al., 2012). So, do different ego depletion initiation task types produce the same effect size? Which ego depletion initiation task produces a more pronounced effect size? No relevant research has been done to date. The power model of self-control suggests that all self-control behaviors share the same resource pool, i.e., ego depletion effects should not be influenced by ego depletion initiation tasks, but different ego depletion initiation tasks have different mechanisms of action and produce different effects (Hagger et al., 2010).

### 1.5 Different types of sports performance

Not only did the researchers use multiple types of ego depletion initiation movement, but they also explore different types of movement performance.

#### 1.5.1 Motor performance

Other task types of motor performance include endurance-based motor performance (sit-ups, push-ups, plate support exercises, handgrip squeeze ergometry) (Dorris et al., 2012; Stocker et al., 2018; Mangin et al., 2021; Dallaway et al., 2023; Englert and Wolff, 2015), and goal-based motor performance (free throws in basketball, set serves in tennis free throws in basketball, serving in tennis, darts tasks) (Englert and Bertrams, 2012; Englert and Bertrams, 2016; Gregersen et al., 2017; Wagner and Wieczorek, 2024), reactive sports performance (starting) (Englert and Bertrams, 2014; Englert et al., 2015a,b), and decision-making sports performance (selection of basketball plays) (Furley et al., 2013). According to previous research, fine-tuned aiming sports performance is more likely to produce ego depletion. This is because athletes need to control their breathing, heartbeat, and even the smallest twitches of their muscles to improve accuracy. Ego depletion can lead to a loss of focus and control, which can affect an athlete’s performance (McEwan et al., 2013), but the results are not known when compared to other sports types.

The present study explored the effects of ego depletion on sports performance from the athletes’ perspective, and proposed three hypotheses: H1 confirmed that ego depletion has significant negative effects on sports performance, suggesting that attention needs to be paid to its negative effects; H2 revealed the differences in the amount of effects produced by different ego depletion initiation tasks to provide a basis for H2 reveals the differences in the effect sizes of different ego depletion initiation tasks, providing a basis for avoiding the ego depletion task; H3 the effect sizes of different types of athletic performance affected by ego depletion are different, to provide guidance on which type of athletic performance should be emphasized in the psychological training of athletes. By systematically sorting out the types of tasks that lead to ego depletion in athletes, this study provides a scientific basis for the development of psychological resource management strategies in training and competition to help athletes break through psychological limitations and maximize their athletic potential.

## 2 Methodology

This meta-analysis was systematically reviewed and meta-analyzed according to the 2020 PRISMA list, which is detailed in Supplementary Appendix A. The registration number at PROSPERO is CRD42024561990.

### 2.1 Literature search

This meta-analysis was searched using a computerized search of the following databases: Pubmed, Web of Science, Scopus, EBSCO, Embase, and Cochrane, and its full text was searched using Google Scholar. The search terms included “ego depletion,” “ego energy,” “self-control,” and “self-regulation” with “sports Performance,” and “physical activity,” respectively, “exercise,” “sports,” and “performance,” respectively. The literature search strategy was developed using Boolean operators (“AND” and “OR”) to concatenate subject terms with free terms. The time frame of the search was from the date of creation of the database to March 8, 2024. The formulation of the search strategy, the search terms, and the search time for this study were developed and conducted by two researchers, Yan Xu and Zhongjing Yi, and any differences that arose during the screening process will be discussed and resolved with a third researcher, Liyan Wang.

### 2.2 Inclusion and exclusion criteria

The inclusion criteria for the study conducted in this article were: (i) study population: athletes (An athlete is an individual who participates in sport to improve physical fitness, skill, or athleticism through systematic training with the primary goal of competing (McKinney et al., 2019), selected regardless of gender, sports performance, and level of specialization), free of any psychological or physiological disorders (Examples include anxiety disorders, depression, bone fractures, and coronary heart disease.); (ii) study content: a variety of relevant sports performances; and (iii) selection of literature from the literature after the dual-task experimental paradigm developed by Baumeister et al. (1998). The dual-task experimental paradigm, which is mainly used to study how humans allocate limited cognitive resources and perform when performing two tasks simultaneously, allows for easier observation of inter-task interferences and reveals the mechanisms of cognitive system functioning and the limitations of resource allocation (Baumeister et al., 1998); (iv) Intervention and control: experimental group with ego depletion generation, control group without ego depletion generation; and (v) Outcome indicators: differences in motor performance, motor performance before and after ego depletion. The exclusion criteria were: (i) exclusion of mismatch with the study population and study content; (ii) exclusion of mismatch in experimental design; (iii) exclusion of literature before 1998; and (iv) exclusion of unavailability of the full text, review articles, conference proceedings, duplicated literature, and animal experiments.

### 2.3 Literature screening and data extraction

Literature screening for this meta-analysis was performed by taking the developed search strategy, searching the database, and importing the retrieved articles into EndNoteX9 for literature screening. First, an initial screening of the literature for duplicates, reviews, conference proceedings, animal experiments, studies that did not match, and no dual-task experimental paradigm before 1998 was performed. Second, the articles after the initial screening were downloaded to obtain the full text. Finally, the literature to be included in the meta-analysis was identified. The process was also mapped out as a flowchart and each step was documented. To ensure the final inclusion of publications that meet the criteria without error, they will be cross-checked again by researcher Zhongjing Yi for reorganization and screening.

Data extraction was performed on the final screening of the literature eligible for inclusion, in which the following were extracted: (i) first author, country, and experimental site; (ii) subject characteristics (sample size, age, group); (iii) ego depletion initiation task; and (iv) exercise performance.

### 2.4 Indicators of results

The outcome indicators in this article were also developed by two researchers, Yan Xu and Zhongjing Yi, in consultation with each other. According to previous studies, ego depletion hurts subsequent sports performance (Baumeister et al., 1998). This article focuses on the meta-analysis of athletes’ scores on the effect measures of subsequent sports performance as an outcome indicator in the presence of ego depletion. Subgroup analyses of different ego depletion initiation task initiation types and different types of sports performances were conducted to investigate that different ego depletion initiation tasks would have the same effect on subsequent sports performances with different effect sizes and that different types of athletes were negatively affected by ego depletion with different effect sizes. Standardization was required for the presence of different units for the outcome indicators in the included text. The difference in means between the experimental and control groups was standardized using the Standardized Mean Difference (SMD) in RevMan 5.4 software to eliminate the effect of magnitude by standardization and to make the effect sizes comparable across studies. The resulting values should be presented as negative numbers, and the greater the value of the negative number, the more pronounced the negative impact of ego depletion on the athlete’s sports performance.

### 2.5 Statistical analysis

Meta-analysis was performed using RevMan 5.4 software (Cochrane Library Collaboration, United Kingdom), which is the official Cochrane Collaboration recommended software for systematic evaluation and meta-analysis and has a high degree of compatibility, templated operation, and an intuitively operable method that is more suitable for novices. The analyses included effect size combinations, heterogeneity tests, forest plot generation, and publication bias assessment (funnel plots). Afterward, Egger analysis was performed using Stata 18.0 software to determine publication bias in the article. The original data of the included studies were all continuous variables, so the standardized mean difference (SMD) and its 95% confidence interval (CI) were chosen as the effect scales for the meta-analysis. SMD values are calculated using $d=\frac{X_{1}-X_{2}}{{\mathrm{SD}}_{\mathrm{pooled}}},S\,D_{p\,o\,o\,l\,e\,d}=\sqrt{\frac{\left(n_{1}-1\right)\,S\,D_{1}^{2}+\left(n_{2}-1\right)\,S\,D_{2}^{2}}{n_{1}+n_{2}-2}}$ and 95% confidence intervals are calculated using 95%*CI* = d±1.96⋅*SE*_d_. Heterogeneity was assessed by Cochran’s Q test and the I^2^ statistic, which reflects the degree of inter-study heterogeneity and is judged as follows: 25% low heterogeneity, 50% moderate heterogeneity, and 75% high heterogeneity (Higgins et al., 2003). When inter-study heterogeneity is small (*P* > 0.1 and I 2 ≤ 50%), Meta-analysis using a fixed-effects model (FE) removes unobserved heterogeneity and eliminates individual- or time-level fixed differences by within-group transformations (Within Transformation) or dummy variables (LSDV), and utilizes only within-individual (or within-time) variation to estimate the parameters; conversely, if *P* < 0.1 and I^2^ > 50%, meta-analysis using a random-effects model (RE) allows mixing of within- and between-group information. It improves the estimation efficiency by weighting within-group variation (within-individual variation) and between-group variation (between-individual differences) through generalized least squares (GLS). To deeply explore the effects of other potential factors on the outcome indicators, subgroup analyses of these factors will be conducted. Publication bias in the literature was assessed by funnel plots, and publication bias was considered to exist if the funnel plots showed asymmetry; otherwise, it was considered to be absent. Interpretation criteria for effect sizes were: less than 0.2 was considered a small effect size, around 0.5 was considered a medium effect size, and greater than 0.8 was considered a large effect size (Cohen, 2013). A z-statistic of *P* < 0.05 was used to assess the significance of the overall effect, and if the resulting p-value was not significant, sensitivity analyses would be performed using the Leave-One-Out method.

## 3 Results

### 3.1 Results of the literature search

**FIGURE 1:**
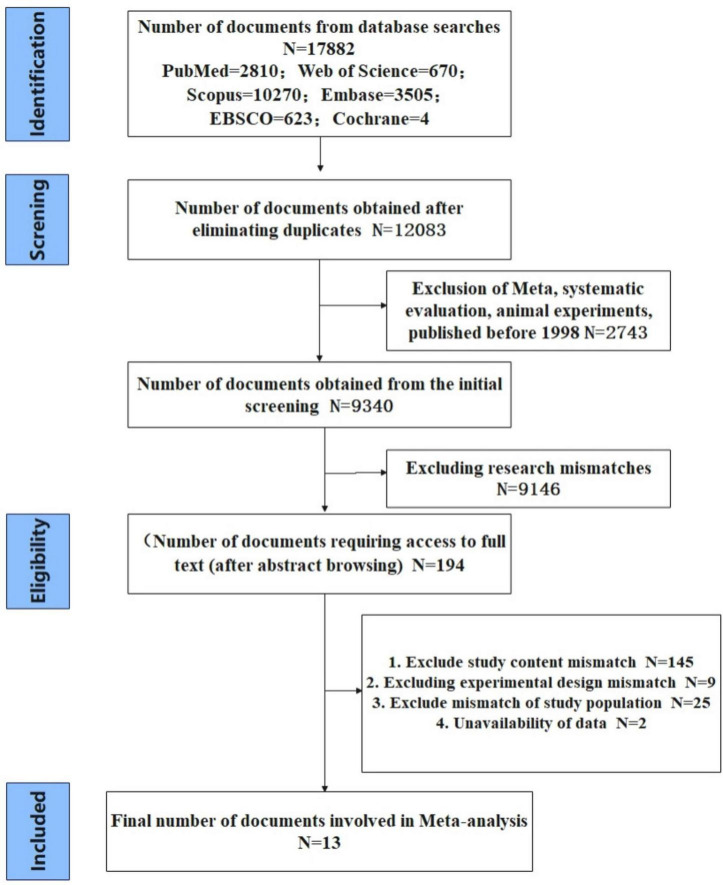
Flowchart of literature screening.

In this article, a joint search of free and search terms in six databases was performed using Boolean operators (was performed using Bototal of 17,882 articles were retrieved, after eliminating those that met the initial exclusion criteria, the initial screening yielded The preliminary screening yielded 194 articles. By reading the title and preface section, experimental design, experimental subjects, study content, and data that could not be extracted were eliminated, and 13 articles were finally included in the meta-analysis. The literature screening process is detailed in Figure 1. However, the total effect size was not significant *P* = 0.4 and intersected with the null line when the initial meta-analysis was performed. Then after sensitivity analysis using the Leave-One-Out method, after analyzing a total of 15 studies from 13 articles by using one-by-one exclusion, the final effect size was significant P = 0.001, and the total effect size did not intersect with the null line, and a total of 12 studies from 11 articles were included.

### 3.2 Basic characteristics of the included studies

First, this article focuses on the analysis of athletes, so the target age of the subjects was chosen to be between 18 and 34 years of age for each sports performance, and a total of 479 athletes were included; second, the inclusion of ego depletion tasks included three Stroop task groups (Wagner and Wieczorek, 2024; Mangin et al., 2021; Dallaway et al., 2023), the transcription task groups (Englert and Bertrams, 2012; Furley et al., 2013; Englert and Bertrams, 2014; Englert and Bertrams, 2015; Englert and Bertrams, 2015; Englert and Bertrams, 2016; Gregersen et al., 2017; Stocker et al., 2018) and maintaining balance plus countdown groups (Dorris et al., 2012). The included articles also addressed aspects of sports performance, where the sports performance included in the articles that underwent meta-analysis included endurance-based sports performance (Dorris et al., 2012; Englert and Bertrams, 2012; Stocker et al., 2018; Mangin et al., 2021; Dallaway et al., 2023), aim-based sports performance (Englert and Bertrams, 2012; Englert and Bertrams, 2015; Englert and Bertrams, 2015; Englert and Bertrams, 2016; Gregersen et al., 2017; Wagner and Wieczorek, 2024), reactive sports performance (starting) (Englert and Bertrams, 2014; Englert and Bertrams, 2015); and finally, the dual-task experimental paradigm, which has been the main research methodology in psychology since 1998, as proposed by Baumeister et al. Therefore, the articles included in the present study are all from 1998 onwards, and selected are also all in English literature. The basic characteristics of the included studies are shown in Table 1.

**TABLE 1:** Basic characteristics of included studies.

Title	Author/year	N	Ego depletion group (N)	Non-ego depletion group (N)	Setting	Country	Age	Ego depletion task	Sports performance
No.1	Dorris et al. (2012) exp1	24	24		Rowers	Irish	Over 18 years old	Balance of tasks	Repress-up
No.1	Dorris et al. (2012) exp2	24	24		Hockey and rugby athletes	UK		Balance of tasks	Sit-up
No.2	Englert and Bertrams (2012) exp1	64	32	32	Amateur male basketball players	German	22.92 ± 6.11	Transcription task	Basketball free-throw task
No.3	Furley et al. (2013)	40	20	20	Basketball players	German	22.85 ± 3.6	Transcription task	Tactical decision making task
No.4	Englert and Bertrams (2014)	37	18	19	Sports students	German	22.05 ± 1.89	Transcription task	Sprint start reaction time
No.5	Englert and Bertrams (2015)	57	19	19	Semi-professional tennis players	German	24.67 ± 4.48	Transcription task	Target areas tennis serves
No.6	Englert and Bertrams (2015)	31	16	15	Experienced male basketball players	German	29.26 ± 4.90	Transcription task	Basketball free-throw task
No.7	Englert and Bertrams (2015)	38	19	19	Female soccer athletes without track and field experience	Switzerland	20.58 ± 2.10	Transcription task	Sprint start reaction time
No.8	Englert and Bertrams (2016)	39	19	20	Experienced basketball players	Switzerland	24.41 ± 2.51	Transcription task	Basketball free-throw task
No.9	Gregersen et al. (2017)	41	20	21	Sports students	Greece	20.02 ± 1.17	Transcription task	Dart task
No.10	Stocker et al. (2018)	34	16	18	Sports students	Switzerland	20.85 ± 1.31	Transcription task	Plank exercise
No.11	Shaabani et al. (2020)	72	18	18	Basketball players	US	28.6 ± 4.0	Stroop color word task	Basketball free-throw task
No.12	Mangin et al. (2021) exp1	188	94	94	Psychology and sports Sciences undergraduates	French	20.42 ± 2.87	Stroop color word task	Muscular endurance test
No.12	Mangin et al. (2021) exp3	51	25	26	Sports sciences recruited from social media young adult students	French	19.84 ± 2.23	Stroop color word task	Muscular endurance test
No.13	Dallaway et al. (2023)	180	30	30	Undergraduate sports and exercise science students	UK	18.79 ± 1.43	Stroop color word task and Stroop number task	Muscular endurance test

### 3.3 Risk of bias assessment

**FIGURE 2:**
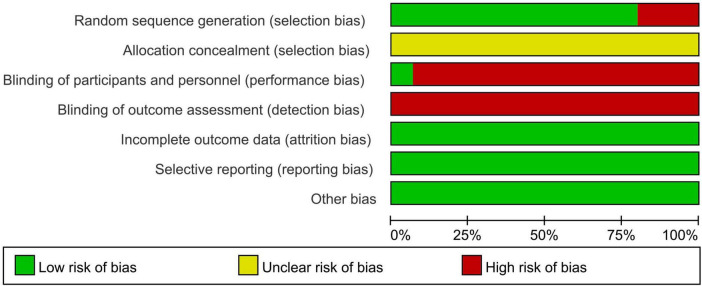
Risk of bias graph.

**FIGURE 3:**
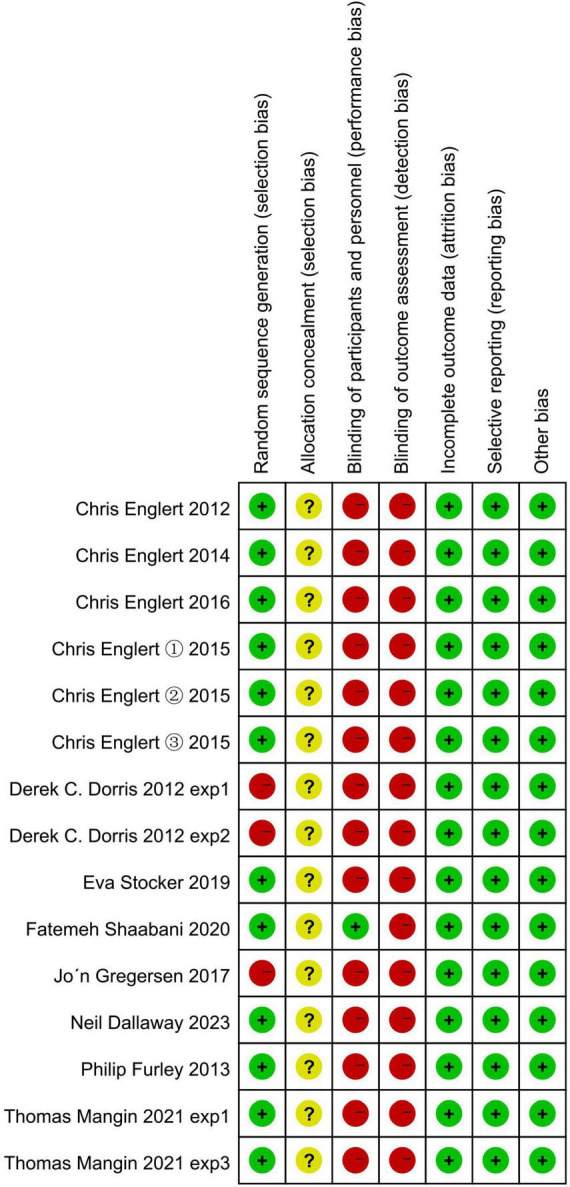
Risk of bias summary.

Using Review Manager 5.4, 12 studies from the 11 articles included in the literature were analyzed for risk of bias assessment. The analysis shows that three of the included studies were non-randomized controlled trials; secondly, whether the subject allocation scheme was hidden or not was mentioned in the original articles; then, only one of the two blinded studies was blinded to both the researcher and the subjects, and only one of them was blinded to the study; and lastly, the completeness of the outcome data, selective reporting, and other biases were all at low risk of bias. The overall risk of bias assessment allows us to conclude that 12 studies from the 11 included papers were at risk of bias. The results are detailed in Figures 2, 3.

### 3.4 Total effect size analysis and heterogeneity assessment

**FIGURE 4:**
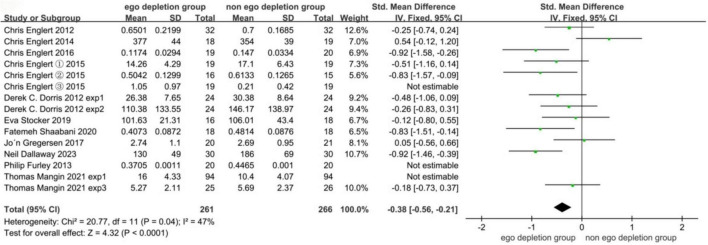
Fixed-effects meta-analysis forest plot results for total effect size.

Through the use of Review Manager 5.4, in the initial meta-analysis, there were experimental results in one of the articles that were not significant; experimental values in one article that had different statistical results (this literature was an upward trend more indicative of the negative effects of ego depletion, which differed from the other studies in the literature) as well as a study that included a sample size that was too large, which caused the article was over-represented, resulting in an overall insignificant analysis. meta-analysis was performed on the remaining 11 articles in the literature totaling 12 studies after performing a sensitivity analysis and eliminating them using a case-by-case elimination method. As can be seen from the forest plot, the heteroscedasticity test I^2^ = 47%, indicating that the true difference in effects caused 47% of the total variation, it can be assumed that there was moderate heterogeneity in this study, and the heterogeneity was not significant, so the fixed-effects model was used for the meta-analysis. The combined meta-analysis showed an effect size of −0.38 (95% CI: −0.56 to −0.21), and the effect size was significant (*Z* = 4.32, *P* < 0.0001) with a medium effect size, the ego depletion group (experimental group) had a significantly lower effect size of 0.38 on subsequent exercise performance than the control group, and a significant effect size. The SMDs in this meta-analysis were all negative, indicating a decreasing effect of the experimental group (ego depletion group) over the control group (non-ego depletion group) on athletic performance. Where the negative sign indicates a decrease, the larger the negative value indicates a greater and more pronounced effect of ego depletion on athletic performance. In the following subgroup analyses the negative values and the negative sign represent the same meaning as above. The results of the fixed effects meta-analysis are detailed in Figure 4.

**FIGURE 5:**
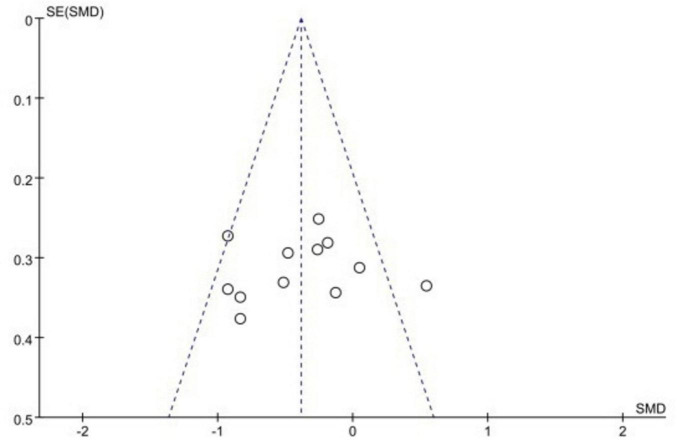
Funnel plots for the test of heteroscedasticity for total effect sizes.

A funnel plot was drawn to examine the publication bias of 12 studies in 11 papers, which is shown in Figure 5. It can be seen from the figure that all the included literature is distributed within the 95% confidence interval, and only one article is excluded, so it can be judged from the funnel plot that there is publication bias in the selected articles. Moreover, all studies are evenly distributed on both sides of the combined SMD. However, the Egger test was performed post hoc using Stata 18.0 software, and the result of *P* = 0.749 (>0.05) indicates that there is no publication bias in the selected articles (for details of the results, see Table 2).

**TABLE 2:** Egger’s test result.

Std_Eff	Coefficient	Std. err.	T	P > |t|	[95% conf. interval]
Slope	−0.0908824	1.125217	−0.08	0.937	−2.598023, 2.416258
Bias	−0.981327	3.650676	−0.27	0.794	−9.11554, 7.152886

### 3.5 Subgroup analysis

In this article, the variable ego depletion was moderated for the initiation task and different exercise types, and subgroup analyses were performed using fixed-effects models.

#### 3.5.1 Startup tasks for ego depletion

**FIGURE 6:**
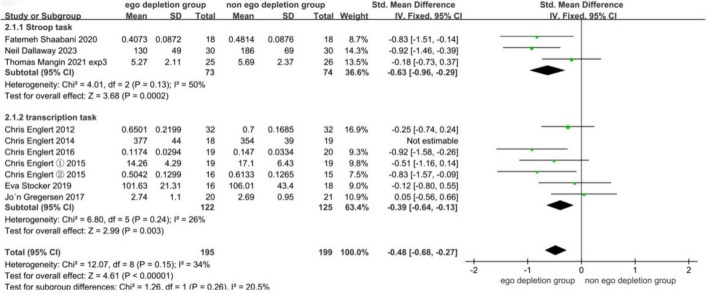
Meta-analysis forest plot results for the ego depletion initiation task.

**FIGURE 7:**
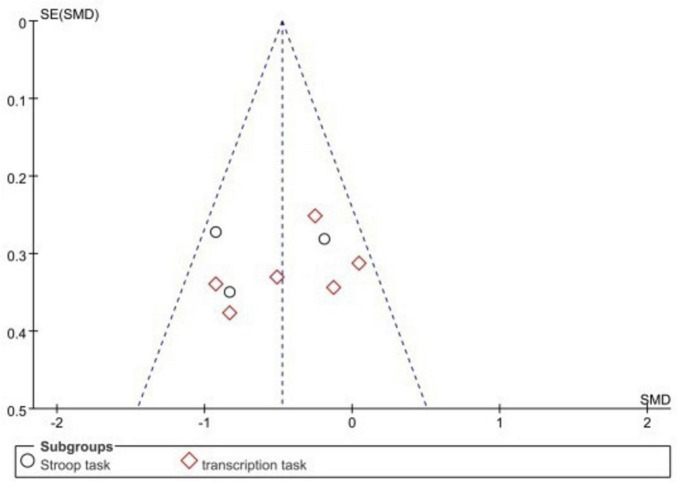
Funnel plots for the ego depletion initiation task.

For meta-analysis, only one experimental study was included in the current subgroup analysis due to maintaining balance plus the inverse group, so it was not included in the current subgroup analysis. One experiment had strong heterogeneity after subgroup analysis, so it was also excluded from the subgroup analysis, as detailed in Figure 6. The results showed that both the transcription task (SMD = −0.39, 95% CI: −0.64 to −0.13, *P* = 0.003) and the Stroop task (SMD = −0.63, 95% CI: −0.96 to −0.29, *P* = 0.0002) all significantly reduced subsequent motor performance, with a moderate overall combined effect size (SMD = −0.48, 95% CI: −0.68 to∼−0.27, *P* < 0.00001), indicating that the experimental group had significantly lower motor performance than the control group. Subgroup comparisons showed a stronger Stroop task effect than transcription task (−0.63 < −0.39). Funnel plots showed that the literature all lies within the 95% CI, as detailed in Figure 7, suggesting no significant publication bias.

#### 3.5.2 Different types of sports performance

**FIGURE 8:**
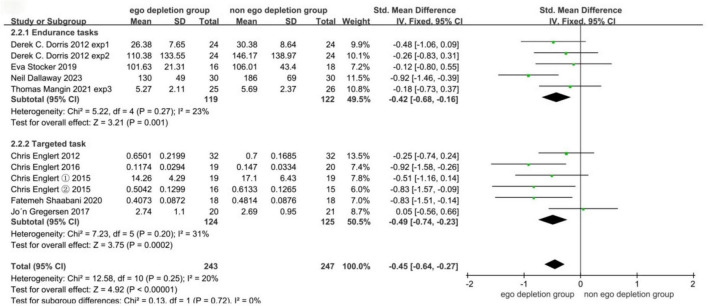
Meta-analysis forest plot results for the different types of motor performance.

**FIGURE 9:**
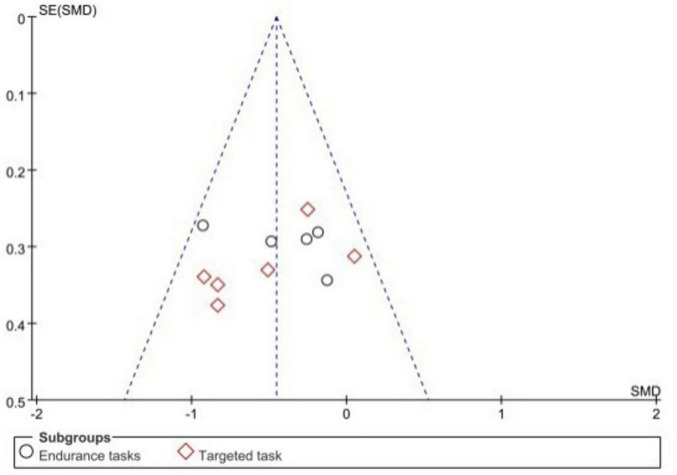
Funnel plots for the different types of sports performances in the ego funnel plots of different sports performance types in the depletion state.

At the time of the meta-analysis, the reactive and tactical decision-making tasks were not included in the current subgroup analysis because there was only one experimental study each. Specific results are detailed in Figure 8. The combined effect size was −0.45 (95% CI: −0.64 to −0.27, *P* < 0.00001), indicating that the ego-depletion group (experimental group) had significantly lower motor performance than the control group. In subgroup analyses, the endurance task effect size was −0.42 (−0.68 to −0.16, *P* = 0.001) and the aiming task effect size was −0.49 (−0.74 to −0.23), and the aiming task was more significantly negatively affected by ego depletion (−0.49 < −0.42). Funnel plots showed that all studies were located within 95% confidence intervals, and the results are detailed in Figure 9.

## 4 Discussion

This meta-analysis focuses on the fact that ego depletion produces a decrease in sports performance, i.e., it validates the hypothesis of the present study, by examining 11 tasks using a dual-task experimental paradigm. It was also verified through subgroup analyses that different ego depletion initiation tasks produce a decrease in sports performance, and that ego depletion produces a decrease in different types of sports performance. According to the interpretation of the self-control strength model, athletes need a certain degree of self-control in situations that produce ego depletion to avoid producing a decline in skill on subsequent sports performance tasks (Hagger et al., 2010). However, based on the total effect sizes in the present meta-analysis, it can be concluded that athletes are unable to overcome prior ego control. According to the explanation of the power model of self-control, i.e., a decline in an athlete’s mental resources can hurt subsequent performance in various sports. That is, athletes are unable to compensate for their missing psychological resources without other means of intervention-initiating effects (Englert and Bertrams, 2015; Englert and Bertrams, 2016; Gregersen et al., 2017; Stocker et al., 2018; Wagner and Wieczorek, 2024), so future studies could also go on to explore interventions for ego depletion.

### 4.1 The effect of ego depletion on sports performance is a medium effect size with possible publication bias

Previous research has demonstrated that ego depletion produces a downward effect on sports performance (Dallaway et al., 2023). Ego depletion not only affects professional athletes but also has a significant effect on college athletes. In the case of ego depletion, there is also an effect on subsequent golf putting (Shin et al., 2019), dart aiming (Yang et al., 2019), handgrip endurance (Graham and Bray, 2015; Alberts et al., 2007), and cycling (Englert and Bertrams, 2015; Englert et al., 2018), among other sports performances, all have negative effects. This shows that ego depletion has the same effect on athletes with different characteristics. Since the experimental subjects selected for this article are mainly athletes to conduct the meta-analysis, according to the forest plot of the overall effect size can be derived from the significant effect size (*Z* = 4.32, *P* < 0.0001), in the meta-analysis derived from the total effect size of −0.38, which has a medium effect size, so the hypothesis of this article is verified. The reason for presenting a result with a medium effect size may be related to the subjects included in the study, according to the study published by Englert et al. which illustrated that ego depletion only has a significant effect on sub-elite athletes who have trained and achieved professionally and are not training systematically at present (Englert et al., 2021). Since the original article did not mention whether or not the subjects studied were sub-elite athletes at the time of inclusion in this article, the results presented had a moderate effect size. The results are still significant overall. As can be seen from the funnel plot, the graph is roughly symmetrical, but one article was excluded. However, after the Egger test, no publication bias was found. The inconsistency between the funnel plot and the Egger test may be related to the small number of included articles. The statistical power of the Egger test depends on the number of studies, and this article only included 12 studies. Secondly, it may also be related to the extreme effect value of this article, which causes the funnel plot to be asymmetric. However, the Egger test may reach different conclusions due to the sensitivity of the regression model to abnormal values. Therefore, there is a possibility that the article has publication bias, and it can be analyzed by including more experimental data in the follow-up.

### 4.2 Different ego depletion initiation tasks produce different effect sizes on sports performance

Although it has been concluded that ego depletion can have a decreasing effect on sports performance, it has not been made to investigate whether the amount of effect on subsequent sports performance would be different for different ego depletion initiation tasks. Therefore, this meta-analysis conducted and explored the first subgroup analysis, and we are the first to analyze the ego depletion initiation task and also the first to analyze a population of athletes. The ego depletion initiation tasks included in this article include the Stroop task (Wagner and Wieczorek, 2024; Mangin et al., 2021; Dallaway et al., 2023) and the transcription task (Englert and Bertrams, 2012; Furley et al., 2013; Englert and Bertrams, 2014; Englert and Bertrams, 2015; Englert and Bertrams, 2015; Englert and Bertrams, 2015; Englert and Bertrams, 2016; Gregersen et al., 2017; Stocker et al., 2018), both yielded significant effect size-standardized mean differences after meta-analysis merging SMD transcription task = −0.39, *P* = 0.003 and SMD Stroop task = −0.63, *P* = 0.0002. It can be concluded that the classical Stroop task significantly depletes athletes’ mental resources and that the task requires participants to perform cognitive inhibition to achieve depletion of mental resources, placing high demands on cognitive processing (see below). Processing places a high demand on the heel (Dallaway et al., 2023). Both of these ego depletion initiation tasks have been commonly used in previous research and both require people to inhibit their thoughts and emotions, but no comparisons have been made to date. The Stroop task, a task of color-word agreement and disagreement, requires subjects to respond quickly to an unfamiliar task that requires either language or a choice of words and requires the individual to undergo two unskilled processing procedures to express it (Wagner and Wieczorek, 2024). In contrast, transcription tasks require people to write a passage of text with the omission of a certain stroke, and in the selected text, the transcribed text is familiar to people in their home country, and expression in written form is a common way of life (Englert and Bertrams, 2014). Compared with the Stroop task, the transcription task is more familiar in people’s lives, so the amount of effect produced may also be lower than the Stroop task. Therefore, the mechanism of action and comparison between the Stroop task and the transcription task can be explored in depth in future research. In conclusion, both tasks reduce sports performance and inhibit athletes’ thinking. Avoiding excessive cognitive and inhibitory thinking in athletes before competitive training and competition can lead to good abilities in subsequent sports performance.

### 4.3 Different types of sports performance are affected by ego depletion with different effect sizes

The second subgroup analysis of this meta-analysis explores whether there is a difference in the amount of effect of ego depletion on different sports performance types. To the best of our knowledge, we are the first to analyze the type of sports performance and the first to analyze a population of athletes. The subgroup analyses revealed that after meta-analysis of the combined SMD _total_ _effect_ = −0.45, both produced a decrease in performance. For aiming-based sports performance, precise aiming maneuvers require a high degree of self-control, but there are often many athletes who are unable to control their ego’s mental activity under the influence of both intrinsic psychological and extrinsic factors, resulting in ego depletion and affecting subsequent sports performance (Englert and Bertrams, 2012; Englert and Bertrams, 2015; Englert and Bertrams, 2015; Englert and Bertrams, 2016; Gregersen et al., 2017; Wagner and Wieczorek, 2024). There is also a significant impact on endurance sports performance, with many athletes unable to tolerate the physical fatigue of high-intensity training and competition and developing thoughts of giving up, resulting in a state of ego depletion (Dorris et al., 2012; Englert and Bertrams, 2012; Stocker et al., 2018; Mangin et al., 2021; Dallaway et al., 2023). There were significant effect sizes for both aiming sports performance and endurance sports performance SMD _endurance_ _task_ = −0.42, p = 0.001 and SMD _aiming_ _task_ = −0.49, *p* = 0.0002, which leads to the conclusion that ego depletion has a greater effect on aiming sports performance and that refined aiming sports performance is more likely to produce ego depletion A comparison of the effects of ego depletion between aiming and endurance sports performance has never been made, and is briefly discussed here. Aimed sports performance in competition and training requires athletes not only to selectively control attention but also to maintain the ability to focus attention during prolonged repetitive presentations of relevant stimuli, requiring individuals to inhibit stimuli that are disruptive to attention (Englert et al., 2021). This is consistent with the fact that the mechanism of action of both the Transcription Task and the Stroop Task is inhibitory control, so the decline is more pronounced (Yue et al., 2023). The endurance task, on the other hand, is a continuous control and does not have a superimposed effect with inhibitory controls such as the transcription task and the Stroop task (Yue et al., 2023). In the present meta-analysis, the amount of effect will be more pronounced in the presentation of the aiming-type sports performance than the endurance-type sports performance. To summarize, ego depletion has an impact on sports performance, especially on targeting sports performance, which should be more aware of the negative effects of ego depletion. Therefore, in future studies, we can also investigate the different mechanisms and effects of ego depletion on different types of athletic performance. Different types of athletic performance have different requirements for self-control and may have different sensitivities to ego depletion. Such differences may lead to heterogeneity in the combined analyses and may account for the moderate heterogeneity in the present article.

### 4.4 Study strengths and limitations

Based on what has been discussed above, the following limitations of the present study need to be further explored: first, limited by the lack of attention paid to the athlete population in the field of sport psychology, the sample size of the original studies included in the analysis was small, which may have affected the statistical efficacy and the stability of the effect sizes. Second, there was potential heterogeneity among the included studies at the experimental design level, as evidenced by the lack of uniformity in the elicitation paradigm of the ego depletion task (e.g., a mixture of cognitive inhibition task and emotion regulation task), and the large variability in the task durations (5, 10, 20 min) and cognitive load intensities, which may interfere with the homogeneity of the results of the meta-analysis. Third, the existing subgroup analyses only roughly classified the types of sports performance, failed to deeply explore the differential response of different sports subcategories (e.g., endurance vs. precision) to the ego depletion effect, and did not examine the moderating relationship between athlete-specific characteristics (e.g., skill level, number of years of training) and the ego depletion effect, which resulted in the limitations of extrapolating the findings to specific sports scenarios. This leads to limitations in extrapolating the findings to specific sports scenarios.

Based on the findings of this article, we can conclude that athletes who produce ego depletion affect subsequent athletic performance. The two means of generating ego depletion in the article are the Stroop task and the transcription task, and their mechanism of action is to achieve depletion of mental resources by inhibiting the athlete’s thinking (Dorris et al., 2012). In practical sports, such as tactical judgment and decision making (judging the trajectory of the next move) (Furley et al., 2013). Similar to the mechanism of action of the Stroop task and the transcription task, athletes are faced with multiple decisions when judging the next move. Still, only one decision is appropriate to be represented in the present moment of motor performance (Furley et al., 2013). During this time, the athlete also needs to inhibit his/her mind to transmit the current game situation to the brain to be analyzed and choose one of the many decisions to be represented (Furley et al., 2013). Therefore, in actual competitions, athletes should not do too many decision-making tasks during the preparation for the competition to avoid increasing their mental burden, which may lead to ego depletion. This still needs to be studied in depth by researchers.

To address the above shortcomings and practical applications, future research can be advanced in three ways: first, in terms of expanding sample representativeness, it is recommended to extend the study to college athletes and youth competitive groups and to establish a multi-center collaborative mechanism to obtain large-sample longitudinal data. Second, the standardized control of experimental design should be strengthened, through the development of evoked intensity grading criteria for ego depletion tasks (e.g., using the MET cognitive load scale to quantify the difficulty of the task), and systematically examining the differential effects of different durations (5, 10, 20 min), and types of tasks (cognitive/emotional/behavioral inhibition) on the athletic performance. In addition, a framework for sport-specific analysis needs to be constructed, in which on the one hand, exercise programs can be finely categorized according to energy metabolism characteristics (aerobic/anaerobic) and skill structure (open/closed skills), and on the other hand, neurophysiological indices (e.g., heart rate, electroencephalogram, electromyography, blood serum cortisol, blood glucose concentration, and blood lactate threshold) may be introduced to assist in evaluating the neural mechanisms of ego depletion. Finally, efforts should be made to develop targeted intervention programs, such as reassessment training on the negative cognitive effects of ego depletion through positive thinking, listening to music, self-talk, etc., to establish a dual-path model of ego depletion prevention intervention in athletic contexts.

## 5 Conclusion

This meta-analysis reveals that ego depletion can have a potentially decreasing effect on athletes’ subsequent sports performance in the domain of sports and that there is a moderate effect size. Subgroup analyses revealed that different ego depletion initiation tasks had different effect sizes on athletes’ performance and that the Stroop task was more likely to cause ego depletion in athletes than the Transcription task. The effect sizes of different types of sports performance affected by ego depletion also varied, with the Aiming sports program performance having a more pronounced negative effect on ego depletion than endurance sports program performance. The present meta-analysis verified the three hypotheses I proposed. A warning is provided for athletes and coaches to avoid producing inhibitory ego depletion initiation tasks, such as scheduling more than 2 high cognitive load tasks in a row, before future competitions and training, especially for tactical case analysis, to avoid excessive ego depletion in athletes. more attention from coaches is needed for future training scheduling for all types of Sports performance types provide a reference model and warning to avoid ego depletion.

## Data Availability

The original contributions presented in the study are included in the article/Supplementary material, further inquiries can be directed to the corresponding authors.
