# Supplementary material for: How does ego depletion reduce sports performance in athletes? A systematic meta-analysis
Source: Front Psychol. 2025 Jun 16;16:1528263. doi: 10.3389/fpsyg.2025.1528263 (PMC12206732; doi:10.3389/fpsyg.2025.1528263)
Supplement: Supplementary file 3 [file Table_3.DOCX]

**Appendix B. Search strategy.**

1. **Search Strategy for PubMed:**

| **Layer** | **Set** | **Search Terms** | **Results** | **Time** |
| --- | --- | --- | --- | --- |
| #1 | ego depletion | ((("ego depletion"[Title/Abstract]) OR ("ego energy"[Title/Abstract])) OR ("self control"[Title/Abstract])) OR ("self regulation"[Title/Abstract]) | 18917 | 6:29:36 |
| #2 | Sport Performance | (((("Sport Performance"[Title/Abstract]) OR ("physical activit"[Title/Abstract])) OR (exercise[Title/Abstract])) OR (sport[Title/Abstract])) OR (performance[Title/Abstract]) | 1698382 | 6:30:14 |
| #3 | #1 AND #2 |  | 2810 | 6:30:23 |

1. **Search Strategy for Web of Science:**

| **Layer** | **Set** | **Search Terms** | **Results** | **Time** |
| --- | --- | --- | --- | --- |
| #1 | ego depletion | (((TS=(“ego depletion”)) OR TS=(“ego energy”)) OR TS=(“self control”)) AND TS=(“self regulation”) | 1900 | Fri Mar 01 2024 19:45:30 GMT+0800 |
| #2 | Sport Performance | ((((TS=(“Sport Performance”)) OR TS=(“physical activit”)) OR TS=(exercise)) OR TS=(sport)) OR TS=(performance) | 6711153 | Fri Mar 01 2024 19:47:02 GMT+0800 |
| #3 | #1 AND #2 |  | 670 | Fri Mar 01 2024 19:47:06 GMT+0800 |

1. **Search Strategy for Scopus:**

| **Layer** | **Set** | **Search Terms** | **Results** |
| --- | --- | --- | --- |
| #1 | ego depletion | ( TITLE-ABS-KEY ( "ego depletion" ) OR TITLE-ABS-KEY ( "ego energy" ) OR TITLE-ABS-KEY ( "self control" ) OR TITLE-ABS-KEY ( "self regulation" ) ) | [55,459](http://www-scopus-com-443.zjnl.tsg211.com/search/history/results.uri?origin=searchhistory&shid=1) |
| #2 | Sport Performance | ( TITLE-ABS-KEY ( "sport performance" ) OR TITLE-ABS-KEY ( "physical activit" ) OR TITLE-ABS-KEY ( exercise ) OR TITLE-ABS-KEY ( sport ) OR TITLE-ABS-KEY ( performance ) ) | [8,778,183](http://www-scopus-com-443.zjnl.tsg211.com/search/history/results.uri?origin=searchhistory&shid=2) |
| #3 | #1 AND #2 | ( ( TITLE-ABS-KEY ( "ego depletion" ) OR TITLE-ABS-KEY ( "ego energy" ) OR TITLE-ABS-KEY ( "self control" ) OR TITLE-ABS-KEY ( "self regulation" ) ) ) AND ( ( TITLE-ABS-KEY ( "sport performance" ) OR TITLE-ABS-KEY ( "physical activit" ) OR TITLE-ABS-KEY ( exercise ) OR TITLE-ABS-KEY ( sport ) OR TITLE-ABS-KEY ( performance ) ) ) | [10,270](http://www-scopus-com-443.zjnl.tsg211.com/search/history/results.uri?origin=searchhistory&shid=3) |

1. **Search Strategy for EBSCO:**

| **Layer** | **Set** | **Search Terms** | **Results** | **Time** |
| --- | --- | --- | --- | --- |
| S1 | ego depletion | TI “ego depletion” OR TI “ego energy” OR TI “self control” OR TI “self regulation” | 15,390 | Friday, March 01, 2024 12:24:32 PM |
| S2 | Sport Performance | TI “Sport Performance” OR TI “physical activit” OR TI exercise OR TI sport OR TI performance | 988,810 | Friday, March 01, 2024 12:24:32 PM |
| S3 | S1 AND S2 | (TI “Sport Performance” OR TI “physical activit” OR TI exercise OR TI sport OR TI performance) AND (S1 AND S2) | 623  397（重）  226 | Friday, March 01, 2024 12:24:32 PM |

1. **Search Strategy for Embase:**

| **Layer** | **Set** | **Search Terms** | **Results** | **Time** |
| --- | --- | --- | --- | --- |
| #1 | ego depletion | 'ego depletion':ab,ti OR 'ego energy':ab,ti OR 'self control':ab,ti OR 'self regulation':ab,ti | 21466 | 1 Mar 2024 |
| #2 | Sport Performance | 'sports performance':ab,ti OR 'physical activity':ab,ti OR 'exercise':ab,ti OR 'sport':ab,ti OR 'performance ':ab,ti | 2206080 | 1 Mar 2024 |
| #3 | #1 AND #2 |  | 3505 | 1 Mar 2024 |

1. **Search Strategy for Cochrane:**

| **Layer** | **Set** | **Search Terms** | **Results** | **Time** |
| --- | --- | --- | --- | --- |
| #1 | ego depletion | ego depletion | 42 | 08/03/2024 13:05:27 |
| #2 | ego depletion | （ego energy）：ab，ti，kw OR （self control）：ab，ti，kw OR （self regulation）：ab，ti，kw | 0 | 08/03/2024 13:05:27 |
| #3 | #1 or #2 |  | 42 | 08/03/2024 13:05:27 |
| #4 | Sport Performance | Sport Performance | 5286 | 08/03/2024 13:05:27 |
| #5 | Sport Performance | （physical activit）：ab，ti，kw OR （exercise）：ab，ti，kw OR （Sport）：ab，ti，kw OR （performance）：ab，ti，kw | 0 | 08/03/2024 13:05:27 |
| #6 | #4 or #5 |  | 5286 | 08/03/2024 13:05:27 |
| #7 | #3 AND #6 |  | 4 | 08/03/2024 13:05:27 |
